# Weigh Biomaterials by Quantifying Species-specific DNA with Real-time PCR

**DOI:** 10.1038/s41598-017-05083-9

**Published:** 2017-07-06

**Authors:** Rong Chen, Jiayu Wang, Yi Yuan, Yun Deng, Xianrong Lai, Feng Du, Juan Dong, Xin Huang, Xin Cui, Zhuo Tang

**Affiliations:** 1 Natural Products Research Center, Chengdu Institute of Biology, Chinese Academy of Sciences, Chengdu, 610041 P. R. China; 2 Ethnomedicine College, Chengdu University of Traditional Chinese Medicine, Chengdu, 611137 P. R. China; 3 Pharmacy College, Chengdu University of Traditional Chinese Medicine, Chengdu, 611137 P. R. China; 4 University of Chinese Academy of Sciences, Beijing, 100049 P. R. China

## Abstract

What’s on the label is not what’s in the bottle, from food products to herbal medicinal products (HMPs), economically-motivated biomaterials adulteration is a long-term problem affecting the food and drug industry. Accurate identification of the biomaterial ingredients in processed commodities is highly desirable. In this field, DNA-based techniques have proved to be powerful tools to overcome qualitative challenges. However, is it possible to quantify the weight of biological materials with PCR? Therefore, a basic scientific question needs to be answered: what’s the relationship between DNA content and the mass of biological materials? Is DNA content directly proportional to the mass of biological materials as most of the researchers previously thought? In this study, we firstly found that there exists a linear relation between DNA contents and the weight of biomaterials indeed when the analytical practices are fully controlled. In this case, the mass of targeted biomaterials in the highly processed commercial products can also be calculated by quantifying the species-specific DNA through classic real-time PCR with a good reproducibility.

## Introduction

Can we weigh biomaterials with other methods rather than analytical balance? Biomaterials are the main sources for food and drugs. And the adulteration in food and drugs (especially in herbal drugs) is a universal problem around the world. If food adulteration is only a matter of integrity or honesty, the herbal medicines adulteration could cause a more serious problem since it can directly reduce the therapeutic effect of the original drug^1^ or even pose a serious risk to the health of the consumers^2^. For instance, in Belgium during 1990–1992, the toxic herb *Aristolochia fangchi* was misused as *Stephania tetrandra* in a diet drug and resulted in over one hundred women’s renal failure. Hence, it is of great importance to establish a scientifically based quality evaluation system for biomaterials in processed herbal medicines. Generally, the authentication and quantification of herbal products mainly rely on chemotype-driven analytical techniques, but this kind of chemical methods often meet some challenges, as the target compounds may not be the specified chemicals with clinical effects, or they also present in other plants. Therefore, in the past few years, DNA-based molecular biology techniques are gaining sharply acceptance and popularity in the qualitative identification of biological materials as DNA is more species specific and less negatively influenced by environmental conditions than chemical constituents^3–6^. However, most of the studies focused on the quality identification, only a few studies have used the PCR based method to quantify biomaterials like meats^7^ or genetically modified products^8^. We can’t help asking that whether the weight of biomaterials can be weighed by PCR based methods? A basic scientific question needs to be answered: what’s the relationship between DNA content and the mass of biological materials? We generally think that the heavier the sample is, the greater cell number is in the sample, involving more DNA content in the sample. In the previous studies^7, 8^ mentioned above, they quantitatively analyzed the biomaterials with real-time PCR by assuming that the DNA content is proportional to the weight of biomaterials without further experimental evidence. To the best of our knowledge, no research has reported the exact relationship between DNA content and the weight of herbal products or any other biomaterials. Only when a good liner relationship is verified or an exact mathematical model between the DNA content and the mass of biological materials is established can we weigh the biomaterials by PCR based technique.

Stigma of *Crocus sativus* L. or saffron has been widely used as medicine, perfume, spice and dye for thousands of years and remains among the most precious plants in the world (the market price for 1 kg of quality saffron now exceeds $5,000)^9^. Due to its high price, saffron is often found to be adulterated with other cheaper materials, such as *Carthamus tinctorius* L., *Nelumbo nucifera* Gaertn, *Zea may* L. and so on. Therefore, we choose saffron crude drugs and saffron contained herbal products to study this basic scientific problem.

## Results

### Comparison of three different DNA extraction methods

Naturally, DNA based quantitative detection methods require a consistent extraction efficiency for the same samples. Nevertheless, most of the current DNA isolation approaches consist of laborious procedures including cell lysis, centrifugation, washing and elution. It is reasonable to think that the more steps, the more system errors could be introduced. Therefore, in this study, we firstly compared three different mainstream DNA isolation methods. Commercially available direct plant DNA extraction solution and silica-based spin column DNA isolation kit showed high repeatability with relative standard deviations (RSD) at 0.6% and 0.7% (Table S2). The traditional CTAB method displayed higher extraction efficiency but lower repeatability with RSD at 5.3% (Table S2), indicating that it is not appropriate for DNA isolation in quantitative assays. As direct plant DNA extraction solution is time-saving and performs outstanding repeatability, most of the experiments were carried out with the direct plant DNA extraction solution unless otherwise noted.

### Development and optimization of the real-time PCR assay

TaqMan technique is one of the sequence specific methods allowing real-time PCR monitoring which has been widely used in many fields^10–13^. In a TaqMan based real-time PCR assay, fluorophore labeled oligonucleotide probe could not be cleaved by the taq polymerase with a 5′–3′ nuclease activity and generate fluorescent signal until it is complementary to the target sequence. The utilization of the TaqMan probe along with the specific primers can achieve higher specificity and accuracy compared with other approaches. Therefore, among the various nucleic acid quantification techniques, the TaqMan probe based real-time PCR was chosen to determine the DNA content in biological materials. Primarily, species-specific primer pairs and probes for *C. sativus* were designed on the basis of Internal Transcribed Spacer (ITS) sequence which has been proved to be a perfect DNA barcoding for the authentication of medicinal plants^14^ and quantification for fungus^15^ (Figure S2).

**Figure 1:**
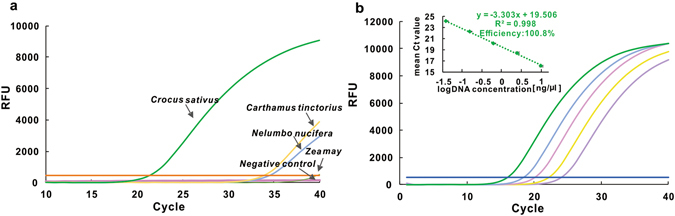
(**a**) Amplification plot generated by *Crocus sativus* and its possible adulterants. (**b**) Amplification plot and calibration curve generated by known concentration of saffron DNA extracts.

Furthermore, real-time PCR conditions including the concentrations of magnesium, probes and DNA polymerase were investigated by orthogonal design experiment. Specificity of the developed real-time PCR was evaluated by 5 real-time PCR reactions, including genomic DNA extracted from same amount of *C. sativus, C.tinctorius, N. nucifera and Z. may*. As shown in Fig. 1a, significant amplification signal was obtained in *C. sativus* reaction with cycle threshold values (Ct values) of 21.36, while no obvious amplification was detected in other species or negative control, with all the ΔCt values >12.

The amplification efficiency of the developed real-time PCR system was determined by analyzing serially diluted DNA extracts (starting DNA concentration 10.1 ng/μl; serial dilution: 1:4, 1:16, 1:64 and 1:256). Figure 1b shows the amplification curves and its corresponding standard curve. R^2^ value of 0.998 in the error bar graph of triplicate PCR reactions successfully demonstrates a good liner relationship between Ct values and the DNA concentration. The slope of the calibration curve is −3.303, corresponding to the amplification efficiency of 100.8%, representing a suitable condition for real-time PCR.

### Validation of the linear relationship between the DNA content and the mass of biological materials

**Figure 2:**
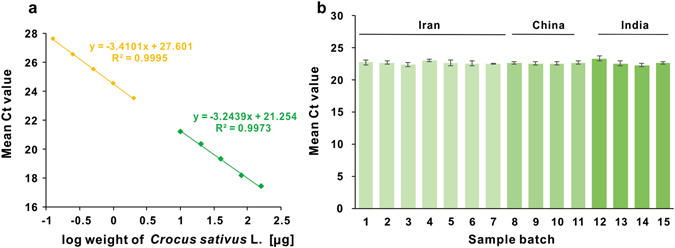
(**a**) Regression curves generated by known amount of *Crocus sativus*. Two regression curves from top to bottom: the DNA was extracted by commercially available direct plant DNA extraction solution (yellow), silica-based spin column DNA isolation kit (green). (**b**) Ct values of saffron samples from fifteen different batch numbers.

It is well known that the basic principle of qPCR (quantitative PCR) relies on the linear relation between the Ct values and the logarithm of the DNA concentration. Therefore, it can be considered that the DNA content is directly proportional to the mass of biological materials and the mass of the biomaterials could be measured by quantifying species-specific DNA with qPCR once a similar linearity relationship between Ct values and the logarithm of the sample weight was also proved. We herein firstly studied the relationship between Ct values and the sample weight. Different amounts of samples (1, 2, 4, 8, 16 mg) were accurately weighed and followed by DNA isolation as well as qPCR analysis. Calibration curve was obtained by plotting the respective Ct values against the logarithm of the sample weight. The correlation coefficients R^2^ value of 0.9995 (DNA extracted by direct plant DNA extraction solution) and 0.9973 (DNA extracted by silica-based spin column DNA isolation kit) successfully demonstrated a linearity between Ct values and the logarithm of the sample weight, which perfectly proved that the DNA content is directly proportional to the mass of biological materials (Fig. 2a).

### Influences of particle size and moisture content on quantitative real-time PCR

To investigate the influence of particle size on the quantitative real-time PCR, we analyzed the saffron samples with different particle sizes. Figure S4 shows that the Ct values of saffron samples fluctuated with the changes of particle size. But for each particle with the same size, the Ct values of three replicates indicated a good repeatability. Therefore, homogenized samples with consistent particle size should be prepared to ensure accurate DNA quantification. It’s also worth mentioning that the particle size of the saffron in herbal products is usually explicitly stipulated by the preparation standard, so this factor would not affect the final analytical results when samples are processed as the description of the standard. As water content can directly influence the quantification results, the moisture content of saffron samples before and after drying were measured by loss-on-drying method. As shown in Figure S5, the moisture content of 10 batch samples varies from 3.56% to 7.55%, but reduces to 2.50–3.02% after drying at 60 °C for 2 hours, showing negligible effect on the repeatability of the Ct values. Therefore, all the samples were dried at 60 °C for 2 hours before the assay in the following experiments.

### DNA content of saffron from different batch numbers

With all the described procedures and factors fully controlled, DNA content of saffron samples from different batch numbers were determined to verify the universality of the developed method. We purchased fifteen batches saffron samples which were produced in China, Iran and India. Although *C. sativus* is mainly cultivated in Iran and India, it was also successfully introduced into many other countries such as Spain, France, China and so on. These saffron samples were grown in different areas with different climate on the earth, but all of them belong to the same species according to DNA sequencing results. The DNA contents in these samples were determined by quantitative real-time PCR. Figure 2b presents the analytical results and the data is evaluated using one-way analysis of variance (ANOVA) (Table S3). The P value is greater than 0.05, which indicates that the DNA content among different batches does not reach statistical significance. Therefore, it can be concluded that the DNA content does not differ from different batch numbers, implying the developed quantification method could have more widespread application prospect.

### Quantitative analysis of saffron in crude drug mixtures

**Figure 3:**
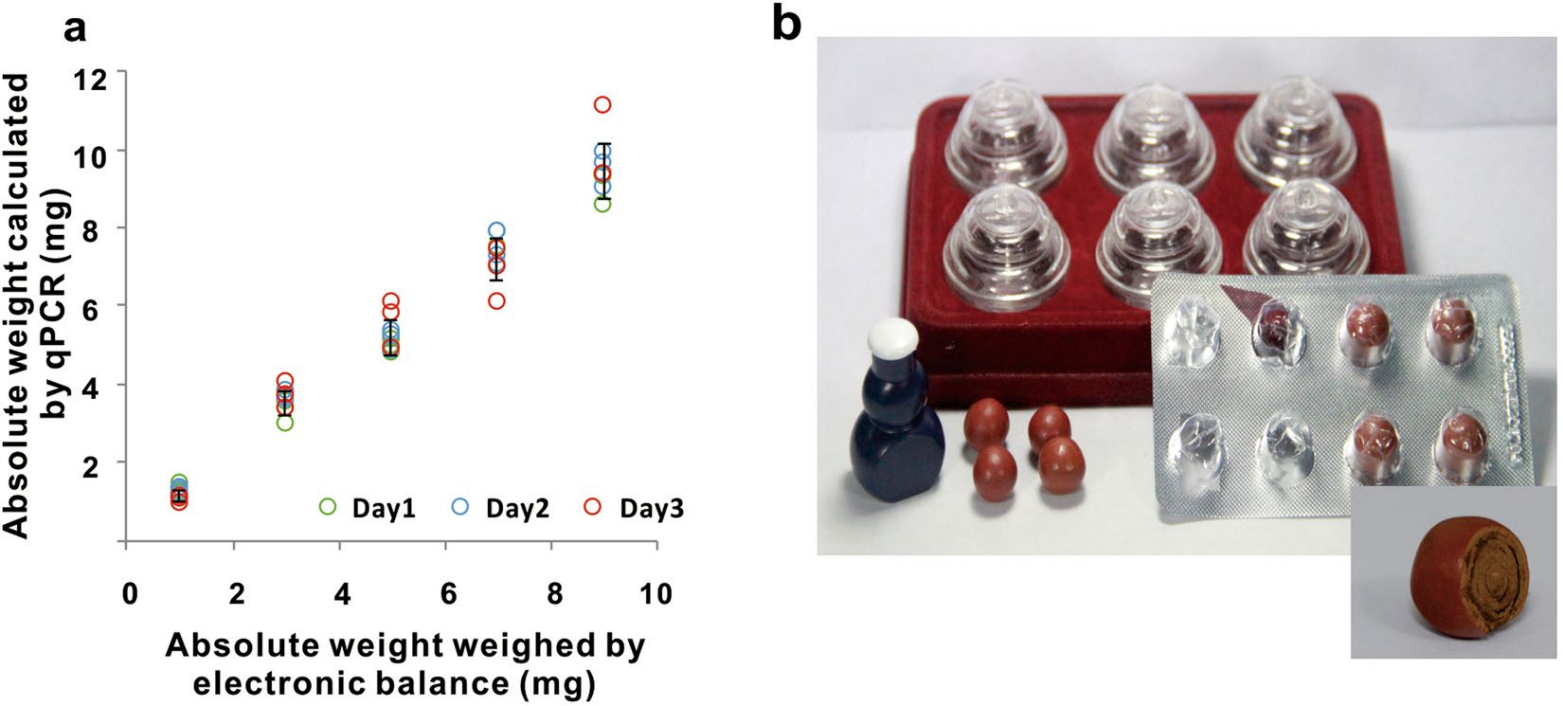
(**a**) Quantitative analysis of saffron in crude drug mixtures. The ratios of saffron to model adulterant in crude drug mixtures are 1 mg/9 mg, 3 mg/7 mg, 5 mg/5 mg, 7 mg/3 mg, 9 mg/1 mg respectively. Each color represents one day (independent weighing and DNA extraction) analytical results (day1, green; day2, blue; day3, red). Error bars represent ± s.e.m. (**b**) Photos of Tibetan medicine “Ershiwuwei Shanhu Wan”.

To verify the feasibility of our method in the quantification of saffron in mixed powder, a series of man-made crude drug mixtures containing different amounts of saffron and model adulterants were prepared **(**Table 1 **)**. The assays of each kind of crude drug mixtures (sampling amount for each assay was 10 mg including 1 mg, 3 mg, 5 mg, 7 mg and 9 mg saffron respectively) were performed in triplicate and repeated for three times on three separate days. It was turned out that the detected saffron in these mixtures were 0.93 mg, 3.02 mg, 5.07 mg, 7.08 mg, 8.84 mg. The relative standard deviations were from 7.44% to 12.77%, revealing that our quantification procedures were efficiently and reliably applied to the quantitative determination of *C. sativus* in crude drugs (Fig. 3a and Table 1).

**Table 1:** Quantitative analysis of saffron in crude drug mixtures-original data of Fig. 3a.

saffron (mg)	model adulterant (mg)	Day1 (mg)			Day2 (mg)			Day3 (mg)			Mean	RSD
		1	2	3	1	2	3	1	2	3	(mg)	(%)
1	9	1.45	1.18	1.24	1.33	1.30	1.19	1.11	1.07	0.93	1.20	12.77
3	7	2.97	3.34	3.52	3.65	3.57	3.83	3.38	3.70	4.07	3.56	8.85
5	5	4.78	4.96	5.13	4.81	5.25	5.34	4.92	5.81	6.10	5.23	8.72
7	3	7.47	7.86	7.05	7.26	7.89	7.05	7.46	6.10	6.97	7.23	7.50
9	1	9.28	9.31	8.59	9.00	9.62	9.91	9.36	11.11	9.35	9.50	7.44

Note: Model adulterants were mixed by equal amount of *C. tinctorius, Z. may* and *N. nucifera* powder.

### Quantitative detection of saffron in complex matrix

**Figure 4:**
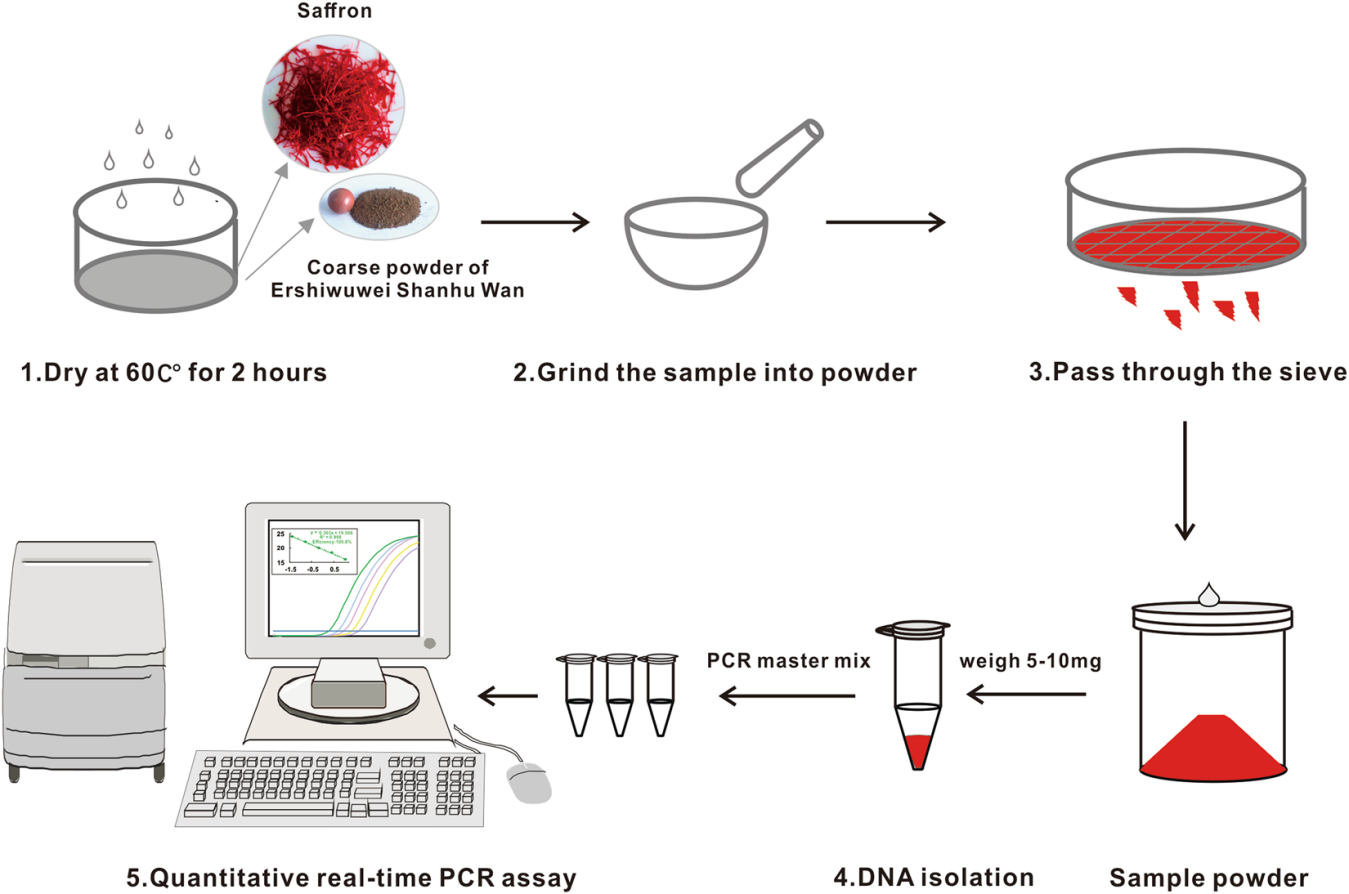
Workflow for quantitative detection of saffron in crude drugs or in Tibetan medicine “Ershiwuwei Shanhu Wan”. Step 1. Dry the saffron samples or coarse powder of “Ershiwuwei Shanhu Wan” at 60 °C for 2 hours to remove excessive water. Step 2. Grind the dried samples into powder. Step 3. Pass the powder through a 100 mesh sieve (saffron powder used for Ershiwuwei Shanhu Wan recovery test was passed through a 200 mesh sieve as it was used in the industrial production) to get homogenized samples. Step 4. Extract the DNA by direct plant DNA extraction solution. Step 5. Determine the saffron in crude drugs or Tibetan medicine “Ershiwuwei Shanhu Wan” by quantifying species-specific DNA through real-time PCR.

Herbal materials are also used in highly processed herbal mixtures. Our previous study demonstrated that PCR is a powerful arsenal for qualitative identification of medicinal materials in processed herbal products^6^. To verify that the developed method can also be applied to the quantitative detection of saffron in complex matrix, a Tibetan medicine named “Ershiwuwei Shanhu Wan” (Fig. 3b) which contains twenty-five kinds of medicinal materials including plants, animals and minerals was analyzed by the developed quantitative real-time PCR. As all the materials are prepared into powders with specified size and mixed together to obtain the final pill, it is an ideal sample to validate the applicability of our method. The contents of saffron in Ershiwuwei Shanhu Wan from three different manufactures were found to be 0.4%, 1.2% and 1.6% respectively **(**Fig. 4 **)**, far below to the requirement of China pharmacopeia (Supporting information 3.7). Therefore, to further validate the accuracy of this method, a recovery test was conducted by adding a certain amount of saffron powder (0.5 mg) to the previous three samples. Then we detected 0.560 mg, 0.568 mg and 0.550 mg of saffron respectively. The corresponding recoveries were from 109.91% to 113.67% **(**Table 2 **)**, clearly proving a high accuracy of the developed method and revealing the feasibility of quantitative detection of saffron in the highly processed herbal products.

**Table 2:** Recovery test for saffron in Ershiwuwei Shanhu Wan.

Sample ID	original (mg/10 mg)	Added (mg)	Found (mg)	△m (mg)	recovery (%)
1	0.04	0.50	0.60	0.56	111.96
2	0.12	0.50	0.69	0.57	113.67
3	0.16	0.50	0.71	0.55	109.91

Note: Recovery [%] = 100 × (amount found-original amount)/amount added; RSD [%] = 100 × SD/mean.

## Discussion

In recent years, the soaring number of quality issues in food and drug industry is a serious problem worldwide^7, 16^. In the food industry, it is estimated that food fraud may cost the global food industry between $10 billion and $15 billion per year, affecting approximately 10% of all commercially sold food products, according to the report of American Grocery Manufacturers Association^17^. While in the area of herbal drug, billions of dollars are spent on herbal medicines or related products every year. Herbal medicines are the main health care resource for people in developing countries and people in developed countries increasingly seek natural products for health enhancement. In the USA, more than half of all adults use dietary supplements to improve their health^18, 19^. In Germany, 90% of the people use natural medicines at some time during their life, whereas in other European countries, the proportion is over 50%^20–22^. But no matter in developing countries or in developed countries, various quality issues of herbal medicines are reported every year^23–25^. That is mainly because most of the herbal materials or herbal products which contain tens of or even hundreds of chemical compounds are too complicated to select one or two compounds to represent the herb itself. Therefore, more and more researchers are coming around to the notion that the current chemistry-based quality control system is hard to completely solve the serious problem that challenges the food and herbal drug industry. So the newly developed DNA-based techniques^6, 26, 27^ are gaining sharply in acceptance and popularity since they provide a simple and reliable way for the authentication of medicinal plants and animals. However, as PCR is a very sensitive method, we could not guarantee the quantity of the biomaterials, especially in some multi-components processed products according to the qualitative PCR techniques. Therefore, in this study, to answer the question whether we can weigh biomaterials by qualification of specific DNA with quantitative real-time PCR or not, we studied the relationship between DNA content and the weight of biomaterials. Our study first proved that there does exist a linear relation between DNA content and the weight of biomaterials. When the analytical practices are fully controlled, we can determine the biological materials by quantifying DNA through qPCR either in the crude biomaterials or in the highly processed commercial products. As the food and herbal drugs involve many kinds of complicated biomaterials, we can expect that the errors of this method will be different according to sample properties but it will also have a broad variety of applications since it can provide a new method to the quality control of food and herbal drug by simultaneously detecting the biological species and quantifying their contents.

## Methods

### Materials

Crude drugs of *Crocus sativus* from different batch numbers were purchased from local farmers in Shanghai, China; herb market in Bozhou, China; herb market in Chengdu, China; local pharmacy in Chengdu, Naqu, WenZhou, China (Figure S1). Crude drugs of *Carthamus tinctorius, Nelumbo nucifera, Zea may* and *C. sativus* contained CPM (sample A) were purchased from local pharmacy in Chengdu, China. The other two commercial *C. sativus* contained CPM (sample B and C) were purchased from local pharmacy in Lhasa, China. To protect the manufacturer’s identities, sample sources were described as letters A-C.

### Sample preparation

Crude drugs of *C. sativus*, *C.tinctorius*, *Z. may*, *N. nucifera* and coarse powder of Ershiwuwei Shanhu Wan were dried in a drying oven at 60 °C for 2 hours and ground into powder in a mortar. All the powder passed through a 100 mesh sieve (*C. sativus* powder used for Ershiwuwei Shanhu Wan recovery test was passed through a 200 mesh sieve as it was used in the industrial production) in order to get homogenized samples for following analysis. Additionally, 1 g each of *C.tinctorius*, *Z. may* and *N. nucifera* powder prepared as described above were mixed together to be used as an experimental model adulterant.

### DNA extraction

Three different DNA extraction methods were used in this study.

1. CTAB method: 10 mg of *C. sativus* powder was accurately weighed and suspended in 750 μl of CTAB buffer (2% CTAB; 100 mM Tris-HCl, pH 8:0; 20 mM EDTA; 2.5 M NaCl) and then incubated at 65 °C for 2 h with occasional shaking. The lysate was extracted with 600 μl of chlorophorm:isoamyl alcohol (24:1). DNA was precipitated with equal volume of 100% isopropanol (30 min at −20 °C, followed by centrifugation at 10,000 g for 15 min). DNA pellet was washed twice with cold 70% ethanol, vacuum dried and resuspended in 100 μl of TE buffer (10 mM Tris-HCl, pH 8:0; 1 mM EDTA).
2. Direct DNA extraction using direct plant DNA extraction solution (Extract-N-Amp™ Plant PCR Kit, Sigma-Aldrich Co. LLC.) was conducted as the manufacturer’s indications with a little modification: a certain amount of (5 mg or 10 mg) each sample was accurately weighed and suspended in 800 μl of DNA extraction solution. The mixture was incubated at 95 °C for 5 min and 10 μl of the lysate was transferred into a new tube. 90 μl of ddH_2_O was added to 10 μl of the obtained lysate and these 100 μl of DNA extracts were used as DNA templates in the qPCR.
3. DNA extraction using silica-based spin column DNA isolation kit (Plant DNA Isolation Kit, Foregene Co., Ltd.) was conducted as the manufacturer’s indications.

### Real-time PCR

Real-time PCR was carried out in a final volume of 20 μl mixture containing 0.25 μl of DNA extracts, 1x taq buffer (20 mM Tris-HCl (pH 8.4), 20 mM KCl, 10 mM (NH_4_)_2_SO_4_ and 5 mM MgSO_4_), 0.2 mM dNTPs, 1.25 units of EasyTaq polymerase and 0.3 μM of each primer and 0.4 μM of probe. All real-time PCR amplifications were conducted under the following cycling conditions: initial template denaturation at 95 °C for 2 min, 40 cycles of 94 °C for 20 sec, 60 °C for 30 sec.

## Electronic supplementary material

Supplementary information
