# Supplementary material for: Weigh Biomaterials by Quantifying Species-specific DNA with Real-time PCR
Source: Sci Rep. 2017 Jul 6;7:4774. doi: 10.1038/s41598-017-05083-9 (PMC5500521; doi:10.1038/s41598-017-05083-9)
Supplement: Supplementary file 1 — Supplementary information [file 41598_2017_5083_MOESM1_ESM.pdf]

Supplementary Information

Weigh Biomaterials by Quantifying Species-specific DNA with Real-time PCR

Rong Chen, Jiayu Wang, Yi Yuan, Yun Deng, Xianrong Lai, Feng Du, Juan Dong, Xin Huang, Xin Cui, Zhuo Tang

Table of contents

1. Material and Apparatus 2

2. DNA Sequences 3

3. Results and Discussion 5

    3.1 Repeatability of three different DNA extraction methods 5

    3.2 Influences of particle size on DNA quantitative analysis 6

    3.3 Influences of moisture content on DNA quantitative analysis 7

    3.4 Quantitative analysis of DNA content in different batches of saffron 8

    3.5 Quantitative determination of saffron in man-made crude drug mixtures 10

    3.6 Recovery test for the saffron in Ershiwuwei Shanhu Wan 13

    3.7 China pharmacopeia standard of "Ershiwuwei Shanhu Wan" 14

1. Material and Apparatus

Crude drugs of *Crocus sativus* from different batch numbers were purchased from local farmers in Shanghai, China; herb market in Bozhou, China; herb market in Chengdu, China; local pharmacy in Chengdu, Naqu, WenZhou, China. Crude drugs of *Carthamus tinctorius, Nelumbo nucifera, Zea may* and *C. sativus* contained CPM (sample A) were purchased from local pharmacy in Chengdu, China. The other two commercial *C. sativus* contained CPM (sample B and C) were purchased from local pharmacy in Lhasa, China. To protect the manufacturer's identities, sample sources are described as letters A-C. The oligonucleotides (Supplementary Table 1) were PAGE-purified except the probe that was HPLC-purified by Sangon Biotech Co., Ltd. (Shanghai, China). Taq DNA polymerase, Taq buffer, and dNTPs were purchased from TransGen Biotech (Beijing, China). The direct plant DNA extraction solution (Extract-N-Amp™ Plant PCR Kit) was purchased from Sigma-Aldrich Co. LLC.. The silica-based spin column DNA isolation kit (Plant DNA Isolation Kit) was purchased from Foregene Co., Ltd. (Chengdu, China). The real-time PCR amplifications were performed in a Funglyn Biotech FTC-3000 Real-Time PCR or a Bio-Rad CFX connect Real Time PCR).

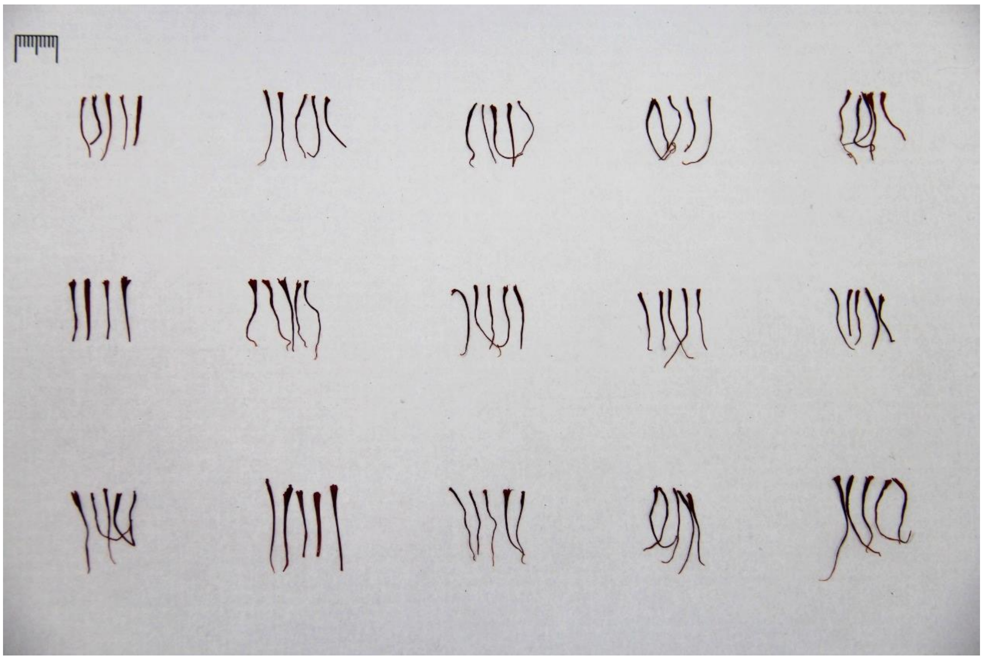

Figure S1. Photos of the 15 batches of saffron samples

2. DNA Sequences

The internal transcribed spacer2 (ITS2) sequence of *C. sativus* (Genbank Accession NO. DQ094185.2), *C. tinctorius* (Genbank Accession NO. JQ230977.1), *N.nucifera* (Genbank Accession NO. AF136290.1), Z. mays (Genbank Accession NO. AF01987.1) were aligned and analyzed by DNAMAN 6.0 (Figure S2). The specific primers and probe were designed on the basis of alignment result by Primer Quest Tool (Integrated DNA technologies) and synthesized by Sangon Biotech (Shanghai, China). All the sequences and final concentrations used in real-time PCR are listed in Figure S2.

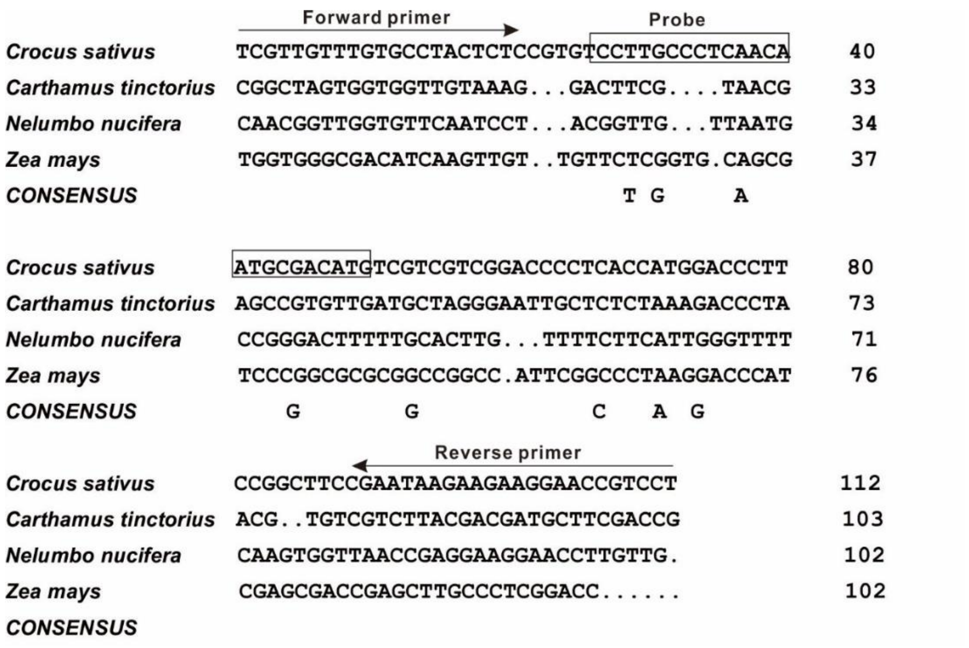

Figure S2. Sequence analysis of the internal transcribed spacer 2 (ITS2), partial sequence. Sequence alignments of the ITS2 from *C. sativus, Carthamus tinctorius, Nelumbo nucifera* and *Zea may*. Primer sequences used for amplification are indicated by arrows and probe sequence is boxed. Dots(.) are introduced for the best alignment.

Table S1. Primers and probe used in the direct real-time PCR assay

Primer/Probe	Sequence	Length (nt)	Final concentration (μM)
forward	TCGTTGTTTGTGCCTACTCTC	21	0.3
reverse	AGGACGGTTCCTTCTTCTTATTC	23	0.3
probe	5'FAM-CCTTGCCCTCAACAATGCGACATG-3'BHQ1	24	0.4

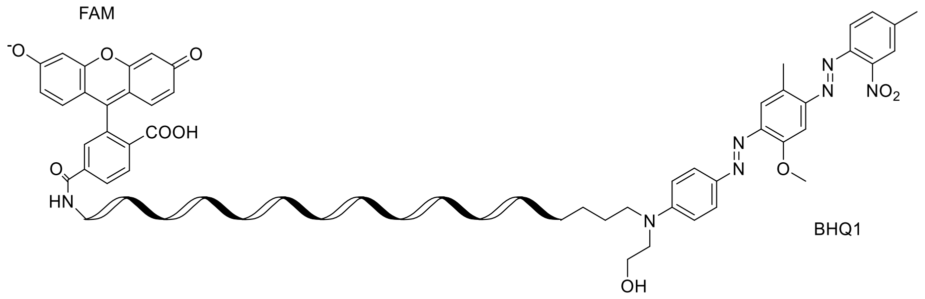

Figure S3. Chemical structures of the Fluorophore and Quencher

3. Results and Discussion

3.1 Repeatability of three different DNA extraction methods

Repeatability of three different DNA extraction methods was calculated by analyzing equal amount of saffron (10mg) DNA extracts in four replicates. Table S2 presents that commercially available direct plant DNA extraction solution and silica-based spin column DNA isolation kit showed high repeatability with relative standard deviations (RSD) at 0.6% and 0.7%. The traditional CTAB method displayed higher extraction efficiency but very low precision with RSD at 5.3%, indicating that it is not appropriate for DNA isolation in quantitative assays.

Table S2. Ct values of saffron samples extracted by three different DNA extraction methods

	Direct plant DNA extraction solution	Silica-based spin column DNA isolation kit	CTAB
Ct value	20.48	17.04	16.18
	20.30	17.28	16.12
	20.20	17.21	17.08
	20.34	17.02	17.98
mean	20.33	17.17	16.46
RSD (%)	0.6	0.7	5.3

3.2 Influences of particle size on DNA quantitative analysis

Influences of particle size on DNA quantitative analysis was investigated by analyzing saffron samples with different particle size. Saffron powder with different particle size (75, 150, 180,250μm) were obtained by passing through four different sieves (200,100,80,65mesh). For each particle size, three equal amount of saffron powder (10mg) were accurately weighed and analyzed by developed real time PCR. It is shown in Figure S4 that from 150-250μm, sample powder with smaller particle size possessed smaller Ct value, indicating a higher DNA extraction efficiency. While from 75-150μm, the particle size might be too small and could inhibit the DNA isolation to some extent. All the error bars in Figure S4 show the standard deviations of three parallel assays. Therefore, homogenized samples with consistent particle size should be prepared to ensure accurate DNA qualification.

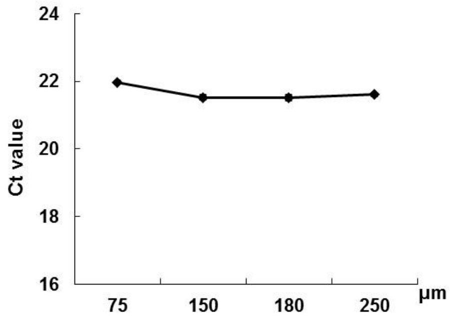

Figure S4. Ct values of saffron samples with different particle size.

3.3 Influences of moisture content on DNA quantitative analysis

The moisture content of 10 batches of saffron samples was measured by loss-on-drying method. Figure S5 shows that the moisture content of 10 batches varies from 3.56% to 7.55%, but after drying at 60℃ for 2 hours, the moisture content reduced to 2.50% - 3.02%. So in order to get uniform samples with consistent water content, we suggest that the samples should be dried at 60℃for 2 hours.

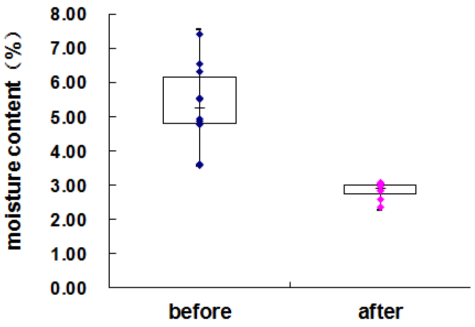

Figure S5. The change of moisture content before and after drying.

3.4 Quantitative analysis of DNA content in different batches of saffron

For each batch of saffron, three parallel samples were accurately weighed (5 mg) and followed by DNA isolation with direct plant DNA extraction solution (Extract-N-Amp™ Plant PCR Kit). The DNA content of each sample was then analyzed in triplicate. Figure S6 shows the detailed schematic diagram of workflow to determine the DNA content in different batches.

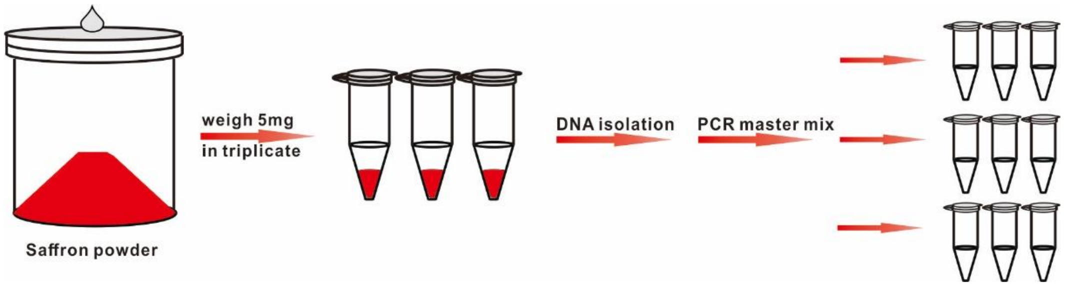

Figure S6. Detailed schematic diagram of workflow to determine the DNA content in different batches of saffron

Table S3. Ct value of saffron samples from different batch numbers (original data of Figure 2B)

Sample batch	origin / place-of-purchase	Ct value			Mean	RSD (%)	*P* value (α=0.5)
1	Iran /Hehuachi herb market,Chengdu,Sichuan,China	22.38	23.02	22.66	22.69	1.43	
2	Iran /local pharmacy store, Wenzhou,Zhejiang,China	22.40	22.63	22.98	22.67	1.28	
3	Iran /local pharmacy store,Naqu,Tibet,China	22.28	22.14	22.77	22.40	1.46	
4	Iran /Hehuachi herb market,Chengdu,Sichuan,China	23.07	22.77	23.22	23.02	1.01	
5	Iran /local pharmacy store,Naqu,Tibet,China	22.15	22.91	22.97	22.68	2.00	
6	Iran/Bozhou herb market,Anhui,China	22.14	22.46	22.91	22.50	1.71	
7	Iran/Bozhou herb market,Anhui,China	22.41	22.51	22.60	22.51	0.42	
8	Shanghai,China/local farmer,Shanghai,China	22.41	22.73	22.75	22.63	0.85	0.0544
9	Shanghai,China/local farmer,Shanghai,China	22.29	22.44	22.83	22.52	1.23	
10	Shanghai,China/local farmer,Shanghai,China	22.52	22.31	22.80	22.54	1.08	
11	Shanghai,China/local farmer,Shanghai,China	22.63	22.41	23.00	22.68	1.31	
12	India/local pharmacy store,Chengdu,Sichuan,China	23.17	23.01	23.78	23.32	1.74	
13	India/Hehuachi herb market,Chengdu,Sichuan,China	22.49	22.19	22.94	22.54	1.68	
14	India/local pharmacy store,Chengdu,Sichuan,China	22.19	22.13	22.55	22.29	1.03	
15	India/Hehuachi herb market,Chengdu,Sichuan,China	22.86	22.62	22.41	22.63	1.00	

3.5 Quantitative determination of saffron in man-made crude drug mixtures

In this section, 1 g each of *C.tinctorius*, *N. nucifera* and *Z. may* powder were mixed together and used as an experimental model adulterant. And then, a series of crude drug mixtures containing 10-90% model adulterants were prepared for quantitative determination.

For each kind of crude drug mixtures, three parallel samples were accurately weighed (10 mg) and followed by DNA isolation with direct plant DNA extraction solution (Extract-N-Amp™ Plant PCR Kit). The weight of saffron in each sample was then analyzed in triplicate by absolute quantification method. For each of the 5 crude drug mixtures studied here, the entire workflow was repeated three times on three separate days. Figure S7 shows the detailed schematic diagram of workflow to determine the saffron in crude drug mixtures.

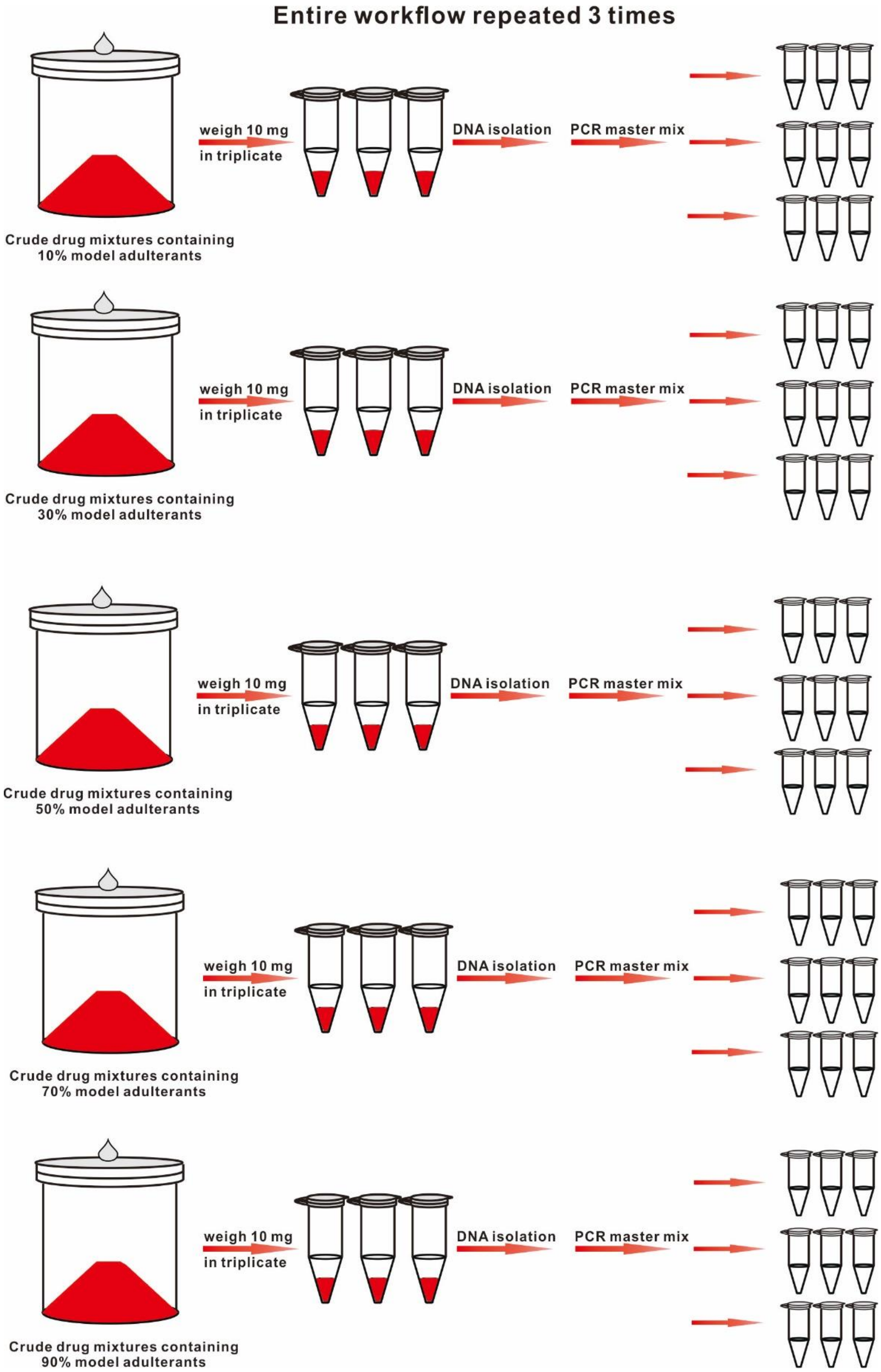

Figure S7. Detailed schematic diagram of workflow to determine the saffron in crude drug mixtures

The calibration curve for absolute quantification analysis was generated by a series of diluted DNA extracts (starting material: the DNA extracts from 10mg of saffron powder; serial dilution: 1:4, 1:16, 1:64 and 1:256). Calibration curve was obtained by plotting the respective Ct values against the logarithm of the weight of saffron. The amplification efficiency (E) was determined by analyzing calibration curves using equation (1). The weight of saffron in an unknown sample can be calculated according to equation (2).

$$E(\%) = [10^{(-1/slope)} - 1] \times 100 \quad (1)$$

$$W\text{（}mg\text{）} = 10\string^[(Ct - d)/slope] \times 8000/1000 \quad (2)$$

3.6 Recovery test for the saffron in Ershiwuwei Shanhu Wan

Firstly, the absolute weight of saffron in Ershiwuwei Shanhu Wan from three different manufacturers was determination in the same way as in crude drug mixtures. Secondly, for each manufacture’s Ershiwuwei Shanhu Wan, 10 mg of each sample was accurately weighed and 0.5mg of saffron powder was added to each sample by subtraction weighing method. And then the DNA was isolated with direct plant DNA extraction solution and followed by the absolute quantification in triplicate.

3.7 China pharmacopeia standard of “Ershiwuwei Shanhu Wan”

Ershiwuwei Shanhu Wan

Ershiwuwei shanhu Pills are prescription used by Tibetan Nationality

Ingredients Os Corallii 75g; Margarita 15g ; Qingjinshi 20g ;Concha Margaritifera 50g ; Fructus Chebulae 100g ;Radix Aucklandiae 60g ;Flos Carthami 80g ;Cloves Flos Caryophylli 35g ;Lignum Aquilariae Resinatum 70g; Cinnabaris 30g ;Os Draconis 40g ; Calamina 25g ;Naoshi 25g ;Magnetitum 25g ;Limonitum 25g ; Semen Sesami 40g ; Fructus Lagenariae 30g ;Flos Asteris 45g ;Herba Swertiae Bimaculatae 80g ;Rhizoma Acori Calami 50g ; Radix Aconiti Kusnezoffii 45g ;Herba Chrysanthemi Tatsiiensis 75g ;Radix et Rhizoma Glycyrrhizae 75g ;Stigma Croci 25g ; Moschus 2g

Procedure Pulverize the above ingredients, except Os Corallii Margarita, Stigma Croci and Moschus, to fine powder and sift, triturate with the above powder. sift, and mix well. Make pills with water, dry in the shade.

Description Redish-brown watered pills; odour, slightly aromatic; taste, sweet, bitter and astringent.

Identification (1) To 0.2g of the powder add 3 ml of hydrochloric acid and 1ml of nitric acid, heat on a water bath for 10 minutes, add 4 ml of water, and filter. To 1 drop of the filtrate add a quantity of copper iodide (mix equal volumes of copper sulfate TS and potassium iodide TS, Eliminate the excess iodine by adding a small quantity of sodium thiosulfate, and use the precipitate), allow to stand, the white precipitate turns to red, To another 1 ml of the filtrate add 0.5 ml of barium chloride TS, a white precipitate is produced.

(2) To 0.5g of powder add 4 ml of methanol, ultrasonicate for 10 minutes, allow to stand, and use the supernatant as the test solution. Prepare a solution with 20 mg ot stigma Croci reference drug in the same manner and use it as the reference drug solution. Carry out the method for thin layer chromatography (Appendix VI B), using silica formic acid and water (5:1:0.8) as the mobile phase. Apply separately 10μL of each of the two solutions to the plate. After developing and removal of the plate, allow to dry in air. The three yellow spots in the chromatogram obtained with the test solution correspond in

position and color to the spots in the chromatogram obtain with the reference drug solution.

(3) Pulverize 3g of the pills to powder, add 10 ml of 80% acetone, ultrasonicate for 30 minutes, filter, use the filtrate as the test solution. Prepare a solution of 0.5g of Flos Carthami reference drug in the same manner as the reference drug solution. Carry out the method for thin layer chromatography (Appendix VIB), using silica gel G as the coating substance and a mixture of ethyl acetate, formic acid, water, and methanol (7:2:3:0.4) as the mobile phase, apply separately 10μl of the above two solutions to air. A major spot in the chromatogram obtained with the test solution correspond in position and color to the spots in the chromatogram obtained with the reference drug solution.

Other requirements Comply with the general requirements for the pills (Appendix I A), except the disintegration time.

Action To promote the restoration of consciousness, blood circulation and relieve pain.

Indications "baimai"disease, unconsciousness, numbness of body, dizziness, headache, abnormal blood pressure, epilepsy and cranial neuralgia.

Usage and dosage 1g, once a day, taken after infusing in boiling water.

Strength (1) 1g per 4 pills (2) 1g per pill

Storage Preserve in tightly closed containers.
